# Ultrastructural alterations and mitochondrial dysfunction in skeletal muscle of peripheral artery disease patients: implications for early therapeutic interventions

**DOI:** 10.17179/excli2024-7592

**Published:** 2024-10-07

**Authors:** Dylan Wilburn, Emma Fletcher, Evlampia Papoutsi, William T. Bohannon, Gleb Haynatzki, Bernd Zechmann, Yuqian Tian, Iraklis I. Pipinos, Dimitrios Miserlis, Panagiotis Koutakis

**Affiliations:** 1 Department of Cell Biology, University of Texas Southwestern Medical Center, Dallas, TX, USA; 2 Department of Public Health, University of West Florida, Pensacola, FL, USA; 3 Department of Surgery, Baylor Scott & White Medical Center, Temple, TX, USA; 4 Department of Biostatistics, University of Nebraska Medical Center, Omaha, NE, USA; 5 Center for Microscopy and Imaging, Baylor University, Waco, TX, USA; 6 Department of Surgery, University of Nebraska Medical Center, Omaha, NE, USA; 7 Department of Surgery, University of Texas Dell Medical School, Austin, TX, USA; 8 Department of Biology, Hal Marcus College of Science and Engineering, Pensacola, FL, USA

**Keywords:** peripheral artery disease, mitochondria, sarcomere atrophy, intramyocellular lipids, sarcoplasm, muscle

## Abstract

Peripheral artery disease (PAD) is an atherosclerotic condition that impairs blood flow to the lower extremities, resulting in myopathy in affected skeletal muscles. Improving our understanding of PAD and developing novel treatment strategies necessitates a comprehensive examination of cellular structural alterations that occur in the muscles with disease progression. Here we aimed to employ electron microscopy to quantify skeletal muscle ultrastructural alterations responsible for the myopathy of PAD. Fifty-two participants (22 controls, 10 PAD Stage II, and 20 PAD Stage IV) were enrolled. Gastrocnemius biopsies were obtained to determine mitochondrial respiration and oxidative stress. Skeletal muscle sarcomere, mitochondria, lipid droplets, and sarcoplasm were assessed using transmission electron microscopy and focused ion beam scanning electron microscopy. Controls and PAD Stage II patients underwent walking performance tests: 6-minute walking test, 4-minute walking velocity, and maximum graded treadmill test. We identified several prominent ultrastructural modifications in PAD gastrocnemius, including reduced sarcomere dimensions, alterations in mitochondria number and localization, myofibrillar disorientation, changes in lipid droplets, and modifications in mitochondria-lipid droplet contact area. These changes correlated with impaired mitochondrial respiration and increased ROS production. We observed progressive deterioration in mitochondrial parameters across PAD stages. Stage II PAD showed impaired mitochondrial function and structure, while stage IV exhibited further deterioration, more pronounced structural alterations, and a decrease in mitochondrial content. The walking performance of Stage II PAD patients was significantly reduced. Our findings suggest that pathological mitochondria play a key role in the skeletal muscle dysfunction of PAD patients and represent an important target for therapeutic interventions aimed at improving clinical and functional outcomes in this patient population. Our data indicate that treatments should be implemented early and may include therapies designed to preserve and enhance mitochondrial biogenesis and respiration, optimize mitochondrial-lipid droplet interactions, or mitigate oxidative stress.

**Translational Perspective**: Peripheral artery disease (PAD) is characterized by skeletal muscle and mitochondrial dysfunction. Ultrastructural changes in skeletal muscle myofibers and mitochondria morphology can provide significant information on the PAD pathophysiology. Here, we investigated skeletal muscle and mitochondria morphological and functional changes at the sarcomere level and across the disease progression and have found that sarcomere lengths and mitochondria count and function are associated with disease progression, indicating loss of skeletal muscle contractile and metabolic function. Ultrastructural changes in the PAD skeletal muscle can provide significant information in the development of new treatments.

## Introduction

Peripheral artery disease (PAD) is a condition characterized by atherosclerotic blockages affecting the arteries supplying the lower extremities (Hiatt et al., 2008[16]; Pipinos et al., 2008[37]). PAD has been classified in four stages according to Fontaine (Koutakis et al., 2018[21]; Norgren et al., 2007[33]). Stage I includes patients that are asymptomatic. Patients presenting with intermittent claudication (IC) are classified as stage II. In Fontaine stage III, PAD patients exhibit foot pain at rest, while the presence of ulcers and gangrene is indicative of stage IV (Koutakis et al., 2018[21]; Norgren et al., 2007[33]). By far, the most common clinical manifestation of symptomatic PAD is intermittent claudication, defined as debilitating leg muscle pain and gait dysfunction induced by walking and relieved by rest (Rutherford et al., 1997[40]). Pain during exercise is caused by restriction of blood flow to the working muscles of the leg (most commonly the calf muscles, but also the thigh and buttock muscles can be involved), creating a painful ischemic environment. In its less common advanced stages, symptomatic PAD becomes limb threatening (known as critical limb threatening ischemia), and patients have foot pain at rest and/or non-healing ulcers and necrosis/gangrene potentially necessitating amputation (Rutherford et al., 1997[40]). While there are currently no cures for PAD, interventions like exercise, revascularization, and pharmacotherapy are available to slow its progression and maintain patient quality of life. 

Although PAD starts as a vascular condition, it progressively impacts the poorly perfused skeletal muscles, with a large amount of data demonstrating the presence of a characteristic myopathy in the legs of patients that contributes to the decline in walking ability (McDermott et al., 2020[29], Pipinos et al., 2007[35], 2008[36]). This myopathy appears to be the product of leg ischemia during walking, followed by reperfusion at rest. In various tissues, including skeletal muscle, a single I/R event can induce the production of reactive oxygen species (ROS) and promote mitochondrial dysfunction (Cadenas, 2018[6]; Kuroda et al., 2020[27]). PAD patients can experience several I/R events daily (essentially every time they walk), initiating a myopathy characterized by muscle atrophy, myofiber degeneration, mitochondrial dysfunction, oxidative damage, inflammation and mitophagy (Casale et al., 2021[7]; Vignaud et al., 2010[42]; Weiss et al., 2013[44]; White et al., 2016[45]). Several studies have elucidated the myopathy-related changes in skeletal muscle in PAD. The preponderance of these investigations have employed light microscopy, while a small subset has used electron microscopy to qualitatively assess the changes in muscle histological structure (Farinon et al., 1984[11]; Hedberg et al., 1988[15]; Koutakis et al., 2015[23][24]; White et al., 2016[45]; Wilburn et al., 2024[47]). However, to the best of our knowledge, there is a paucity of studies which have attempted to evaluate and quantify the ultrastructural changes of myofibers, their contractile elements and their organelles as they occur with the progression of PAD stages and the concomitant development of PAD myopathy. This gap in the literature presents a significant opportunity for advancing our understanding of the pathophysiological mechanisms underlying PAD-related muscle dysfunction. A comprehensive, quantitative analysis of these ultrastructural alterations could potentially elucidate the precise cellular and subcellular changes that occur during disease progression, thereby providing valuable insights into the etiology and potential therapeutic targets for PAD-associated myopathy. 

In this study we aim to quantify major ultrastructural alterations among three cohorts: control subjects, Fontaine stage II PAD patients, and Fontaine stage IV PAD patients. Furthermore, we will investigate whether these structural alterations correlate with two key parameters of PAD myopathy: decreased mitochondrial respiration and increased mitochondrial oxidative stress.

## Materials and Methods

Fifty-two participants (22 controls, 10 Stage II; Fontaine Stage II, 20 Stage IV; Fontaine Stage IV) were recruited and evaluated for lower extremity claudication from PAD in order to take part in this study. The demographics of all patients are listed in Table 1(Tab. 1). Each patient was assessed at the Baylor Scott and White Medical Center (Temple, TX), and the University of Texas Dell Medical School Austin (Austin, TX). Patients' medical histories, ankle-brachial index (ABI), 6-min walking test, maximum treadmill walking test, and 4-meter walking speed were recorded. Muscle biopsies from the center of the medial head of the gastrocnemius were collected to compare the alterations in mitochondrial respiration and ultrastructural features of myofibers. Written informed consent was obtained from each patient who elected to participate. Patients were recruited as part of the human clinical trial NCT04089943 and single-IRB protocol 1624041. All methods implemented comply with the ethical guidelines outlined in the Declaration of Helsinki. 

### Walking performance testing

Each participant completed a 6-minute walk test, a graded treadmill test, and a four-meter walking speed test. During the 6 min walk test, participants were instructed to walk around cones spanning a 20 m distance as often as possible within 6 min. The maximal distance covered within the 6 min was recorded in meters. Two cones were placed 4 m apart to measure four-meter walking velocity, and participants were instructed to walk to the next cone as quickly as possible. The time required to walk the distance was measured and used to determine speed (cm/s). Maximum walking performance was measured for all PAD patients using the graded treadmill test. Briefly, patients walked at a constant speed of 3.2 km/h on a 0° grade that increased 2° every 2 min. Total claudication distance was measured during each graded treadmill test. 

### Mitochondrial respiration

An Oroboros O2k Oxygraph FluoRespirometer (Oroboros Instruments, Innsbruck, Austria) consisting of two temperature-controlled chambers, each containing a polarographic oxygen sensor and a custom-fitted fluorometer (O2k-Fluo LED2 module), was used to simultaneously measure mitochondrial oxygen consumption (*J*O2) and H_2_O_2_ emission (*J*H_2_O_2_) in saponin-permeabilized muscle fibers as previously described (Fletcher et al., 2023[12]; Ismaeel et al., 2022[17]). 

### Transmission Electron Microscopy (TEM)

Muscle samples portioned for structural analysis were immediately fixed and processed as described here (Wilburn et al., 2022[46]). Imaging was conducted using TEM imaging (JEM1010 transmission electron microscope JEOL, Tokyo, Japan). In total, 104 electron micrographs of the intermyofibrillar (IMF) region of myofibers at 2500x magnification (2 per participant) were analyzed, and organelle quantification was carried out using point tracing with the publicly available software Image J (National Institutes of Health, Bethesda, Maryland, USA). All sample preparation and image analysis were conducted, blinded to the condition of each sample until the end of the study. In total, 14,307 mitochondria, 10,865 sarcomeres, and 797 intramyocellular lipids (IMCL) were analyzed by all participants in the study. For each participant total relative mitochondria area, average individual mitochondrial CSA, average mitochondria per Z-disc, and mitochondria count per 250 μm^2^ were measured. Myofibrils were characterized by measuring total relative myofibril area, average m-line length, and Z-disc count per 250 μm^2^. M-line lengths were measured across the sarcomeres from one sarcoplasmic space to the next. Additionally, IMCL total relative area, average individual IMCL CSA, IMCL count per 250 μm^2^, IMCL-mitochondria contact lengths, and total sarcoplasmic area were measured for each participant.

### Focused Ion Beam Scanning Electron Microscopy (FIB-SEM)

A convenience sample of three-participants from the original fifty-two patients were utilized for FIB-SEM. Muscle samples from the control, Fontaine stage II, and Fontaine stage IV patient groups were prepared identically for qualitative mitochondria three-dimensional assessments. The individual resin-embedded samples underwent FIB-SEM milling, serial sectioning, and image collection at 50 nm intervals using the Versa 3D (FEI Company, Hillsboro, OR, USA). The total volume analyzed for each participant was approximately 55 μm^3^. For each participant, 50 serially collected micrographs were aligned using Image J (National Institutes of Health, Bethesda, Maryland, USA), before undergoing manual point tracing for three-dimensional modeling in IMOD (Kremer et al., 1996[26]). All mitochondria and lipid droplets present in the field of view of the serial sections were traced to create iso-surface renderings for qualitative assessment. 

### Statistical analysis

Baseline characteristics of control and PAD Stage II and Stage IV participants were compared using general linear models for continuous variables and chi-square tests for categorical variables to determine confounders. Confounding variables were covariates in subsequent analyses. All electron microscopy, mitochondrial respiration, and oxidative stress variables were statistically assessed group differences by analysis of covariance (ANCOVA) and evaluated post-hoc by Bonferroni adjusted t-tests. Multiple Pearson correlations were conducted to determine if there was an association between the individual mitochondrial function variables and measures of mitochondrial morphology quantified through TEM. Pearson correlations were conducted for mitochondrial respiration, H_2_O_2_ production, and mitochondrial morphology at a significance value of p<0.05. All statistical analyses were performed using GraphPAD Prism 10 (GraphPad Software, Boston, MA, USA).

## Results

### Patient demographics

Data for Control, Stage II, and Stage-IV PAD participants are presented in Table 1(Tab. 1). On average, Stage II and -IV participants had lower diastolic pressure (p<0.0001), higher incidence of coronary artery disease (p=0.025), hypertension (p=0.012), dyslipidemia (p<0.001), and diabetes (p=0.001) compared to control participants. Furthermore, Stage II participants were more likely to be either current or former smokers (p=0.002) compared to controls or Stage IV participants. ABI was significantly decreased with increased disease stage. All of the aforementioned variables were treated as covariates in all subsequent analyses. 

### Walking performance

The Stage II PAD group walked a shorter distance during the 6-minute walking test when compared to the control group (p=0.024; Table 1(Tab. 1)). Similarly, they had significantly shorter maximal walking distance (p=<0.001; Table 1(Tab. 1)) and decreased four-meter walking speed (p=0.001; Table 1(Tab. 1)).

### Mitochondrial morphology, respiration, and oxidative stress

**Figure 1:**
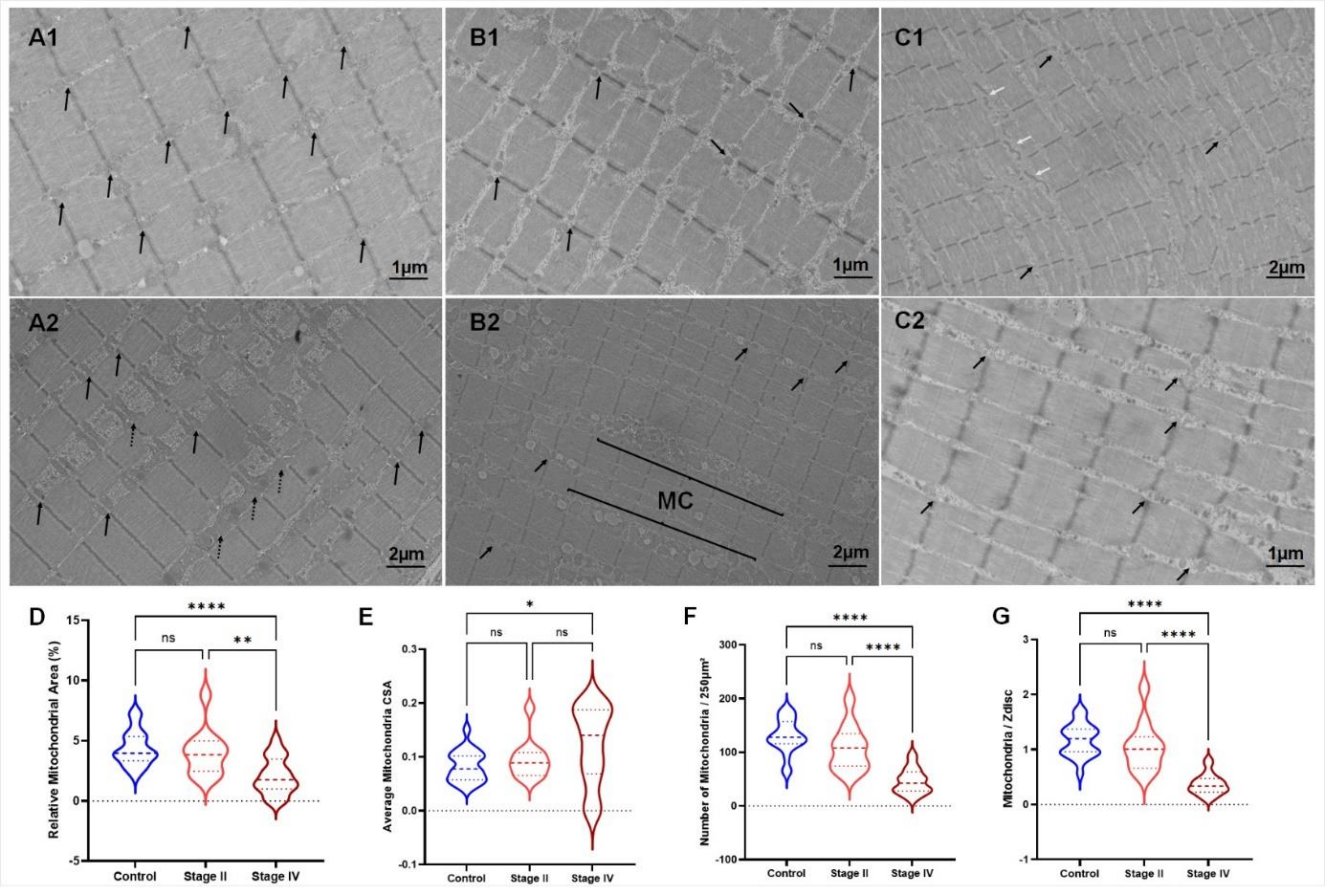
Mitochondrial morphology is abnormal in PAD patients. In control sarcomeres, mitochondria located around the Z-disc (A1-2; black arrows) and in some cases extended throughout the length of the sarcomere and transversely near the locations where a Z-disc would typically be present (A2; dotted black arrow). In Stage II PAD patients (B1-2), there were less mitochondria per Z-disc (B1-2; black arrows) and mitochondrial clusters (MC) were evident (B2). In Stage IV PAD patients (C1-2), there were sparse mitochondria around the Z-disc with some mitochondria being present in the IMF (C1; white arrows). Quantitative measurements (D-G) demonstrated several abnormalities in the mitochondrial morphology in both PAD stages compared to controls.

Qualitative assessments of mitochondrial structure across all groups can be found in Figure 1(Fig. 1). Mitochondria in the control group appear to consistently reside in pairs near the Z-discs of sarcomeres throughout the entirety of the IMF space (Figure 1 A1(Fig. 1); black arrows). In some instances, the mitochondria in the muscle of the control participants extended throughout the length of the sarcomere and transversely near the locations where a Z-disc would typically be present (Figure 1 A2(Fig. 1); dotted black arrow). Although the mitochondria of stage II PAD participants were also mostly found in pairs near the Z-discs (Figure 1 B1(Fig. 1)), certain regional irregularities were noted. For example, specific regions of the IMF space of stage II PAD participants showed irregularly dense mitochondrial clusters (MC), whereas some Z-discs were without a pair of mitochondria (Figure 1 B2(Fig. 1); MC). There were very few mitochondria in the muscle of stage IV PAD participants (Figure 1 C1-2(Fig. 1)). Several mitochondria present within the IMF space of stage IV PAD participants displayed an elongated appearance, which was rarely noted in either the control or stage II PAD participants (Figure 1 C1(Fig. 1); white arrow). 

Quantitative analysis demonstrated that the relative mitochondrial area was reduced in stage IV PAD participants when compared to control (<0.0001) and stage II (p= 0.0052) patients (Figure 1D(Fig. 1)). However, the average mitochondrial CSA (Figure 1E(Fig. 1)) was significantly increased in stage IV participants, but only compared to controls (p= 0.0423). This increase in mitochondrial CSA in stage IV PAD participants was combined with a decrease in the number of mitochondria/ 250 μm^2^ (Figure 1F(Fig. 1)) compared to controls (p<0.0001) and stage II (p<0.0001). There was a significant decrease in the total number of mitochondria/Z-disc (Figure 1G(Fig. 1)) between control and stage IV (p<0.0001), and stage II and stage IV (p<0.0001) PAD participants. 

**Figure 2:**
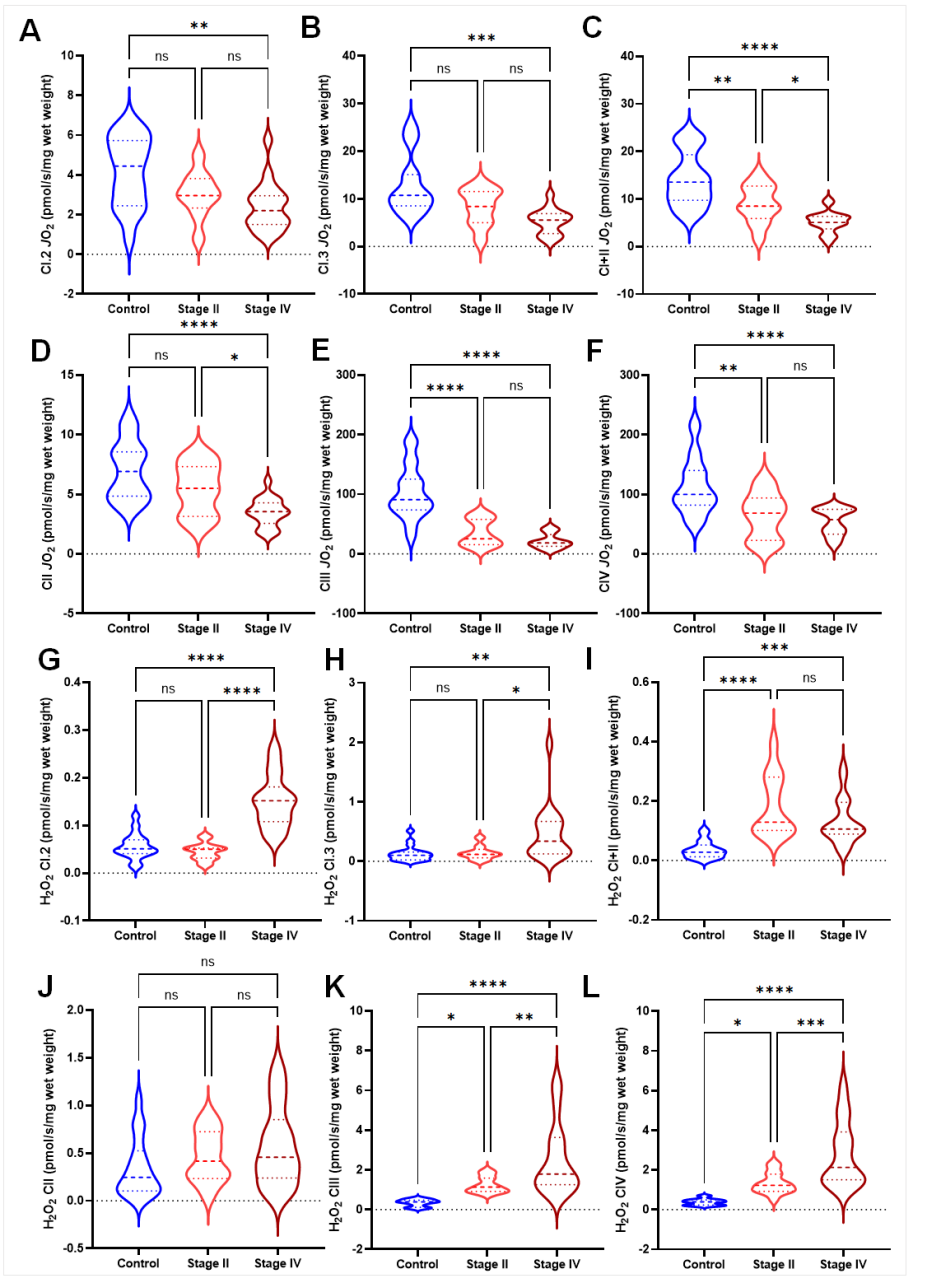
Mitochondrial respiration and mitochondrial H_2_O_2_ production demonstrate significant limitations in PAD groups. Mitochondrial respiration was significantly reduced in several parameters (A-F) within the PAD stages when compared to controls, while H_2_O_2_ production (G-L) was significantly increased.

There was a significant decrease in the average rate of oxygen consumption of stage IV PAD participants compared to controls (p=0.0025) during Complex I state 2 (CI.2) and Complex 1 state 3 (CI.3) respiration (Figure 2A and B(Fig. 2)). When combined, oxygen consumption from complex CI+II (CI+II) was significantly lower with disease progression; i.e., the rate of oxygen consumption was reduced in both stage II (p=0.005) and stage IV (p<0.0001) PAD participants compared to controls (Figure 2C(Fig. 2)); however, oxygen consumption was higher in stage II (p=0.042) compared to stage IV PAD participants (Figure 2C(Fig. 2)). When assessed alone, the rate of oxygen consumption from Complex II (CII) was only reduced in stage IV PAD participants compared to controls (p<0.0001), and stage II (p=0.036) PAD participants (Figure 2D(Fig. 2)). However, the rate of oxygen consumed during Complex III (CIII) was significantly decreased in both stage II (p<0.0001) and stage IV (p=0.0001) PAD participants compared to controls (Figure 2E(Fig. 2)). Complex IV (CIV) oxygen consumption was similarly decreased in stage II (p<0.0001) and stage IV (p=0.0001) PAD participants compared to controls (Figure 2F(Fig. 2)).

Mitochondrial *J*H_2_O_2 _was significantly increased at baseline CI.2 and CI.3 states in stage IV PAD participants compared to controls (p<0.0001 and p=0.004, respectively), and stage IV compared to stage II PAD participants (p<0.0001 and p=0.032, respectively) (Figure 2G-H(Fig. 2)). CI+II demonstrated a significant increase of *J*H_2_O_2_ in both stage II (p<0.0001) and stage IV (p=0.0007) PAD participants compared to controls (Figure 2I(Fig. 2)). No differences were observed for CII (Figure 2J(Fig. 2)). CIII* J*H_2_O_2 _rates were significantly increased in stage II (p=0.045) and stage IV (p<0.0001) PAD participants compared to controls, and stage IV compared to stage II PAD participants (p=0.003) (Figure 2K(Fig. 2)). Similarly, CIV* J*H_2_O_2 _rates were significantly increased in stage II (p=0.028) and stage IV (p<0.0001) PAD participants compared to controls, and stage IV compared to stage II PAD participants (p=0.0005) (Figure 2L(Fig. 2)).

The results of all Pearson correlations (R-values and significance) between mitochondrial morphology values and mitochondrial respiration and oxidative stress values can be found in Table 2(Tab. 2). Briefly, there was a moderate to strong significant positive association between number of mitochondria/250 µm^2^ and mitochondrial oxygen consumption from all the complexes of the electron transport chain (ETC). In contrast, the number of mitochondria/250 µm^2^ were negatively associated with mitochondrial H_2_O_2_ production. Further, relative mitochondria area was positively associated with CI+II, CII, CIII and CIV respiration values and negatively associated with H_2_O_2_ CI+II, H_2_O_2_ CIII and H_2_O_2_ CIV. Interestingly, the average individual mitochondria CSA was negatively associated with CI.3, and CII and positively associated with H_2_O_2_ CI.2, H_2_O_2_ CI.3 and H_2_O_2_ CIV.

**Figure 3:**
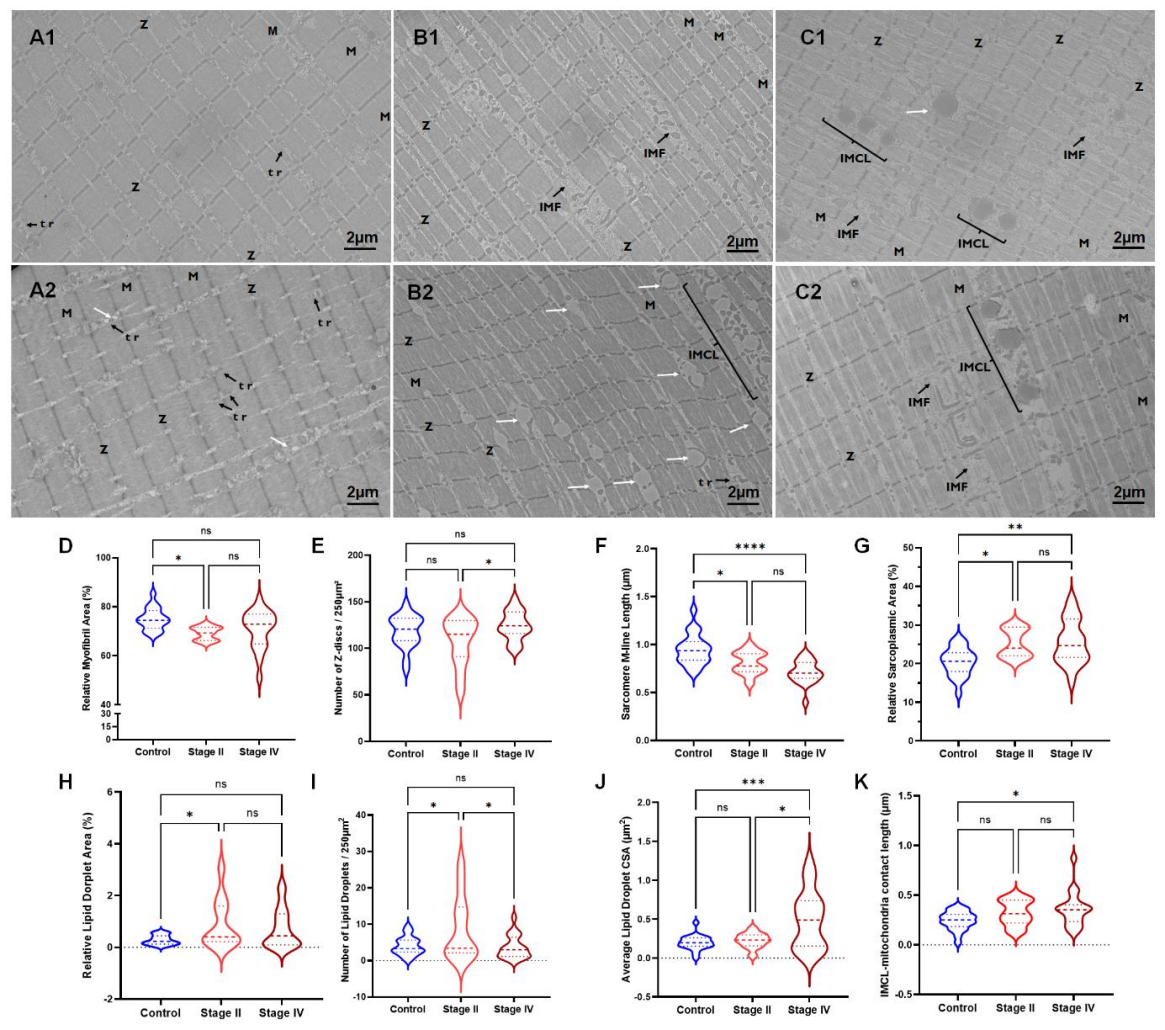
Figure 3 demonstrates the differences in sarcomere changes between control (A1-2), stage II (B1-2), and stage IV (C1-2) PAD patients. M-lines (M), Z-discs (Z), lipids (white arrows), triad (tr; black arrow), and intramyocellular lipids (IMCL). Quantitative measurements of relative myofibril area (D), number of z-discs per 250 μm^2 ^(E), sarcomere M-line length (F), relative sarcoplasmic area (G), relative lipid droplet area (H), number of lipids droplets (I), average lipid droplet CSA (J), and IMCL-mitochondria contact length (K) are demonstrating significant differences between the groups.

Qualitative differences in the sarcomere morphology were apparent across groups. There was a clear difference in the quality of the myofibrils and sarcomeres in the stage II and stage IV patients compared to controls (Figure 3 A1-C2(Fig. 3)). M-lines (M) lengths were shorter in stage II (Figure 3 B1-2(Fig. 3)) and stage IV (Figure 3 C1-2(Fig. 3)) patients and did not have a consistent ridged Z-disc linear structure compared to controls (Figure 3 A1-2(Fig. 3)). Triads (tr) were observed mainly in the controls (Figure 3 A1-2(Fig. 3)) and IMF was increased in stage II (Figure 3 B1-2(Fig. 3)) and stage IV (Figure 3 C1-2(Fig. 3)) patients. IMCL were evident in PAD groups and more prevalent in stage II compared to control and stage IV (Figure 3 A1-C2(Fig. 3); white arrows). Increased IMCL contact length to mitochondria was observed in stage IV PAD patients compared to controls (Figure 3 C1, C2, K(Fig. 3); IMCL).

Quantitative measurements demonstrated a significant decrease in the relative myofibril area between control and stage II PAD groups (p=0.029; Figure 3D(Fig. 3)). A significant increase in the number of Z-discs/250 μm^2^ was found only between stage II and stage IV (p=0.049). Interestingly, both stage II (p=0.023) and stage IV (p<0.0001) PAD patients had significantly shorter sarcomere M-line lengths when compared to control participants (Figure 3F(Fig. 3)). Both stage II (p=0.025) and stage IV (p=0.001) PAD participants showed increased sarcoplasmic areas within the IMF region when compared to control participants (Figure 3G(Fig. 3)). The relative IMF area occupied by lipid droplets was higher in the stage II PAD than controls (p=0.039; Figure 3H(Fig. 3)). The number of lipid droplets/250 μm^2^ was significantly greater in stage II PAD compared to control (p=0.017) and stage IV PAD (0.011) participants (Figure 3I(Fig. 3)). However, stage IV PAD patients had larger lipid droplets than control (p=0.0006) and stage II (p=0.011) participants (Figure 3J(Fig. 3)). Further, there was a significant increase in the mitochondrial-IMCL contact lengths, but only between the stage IV PAD participants and controls (p=0.025; Figure 3K(Fig. 3)).

**Figure 4:**
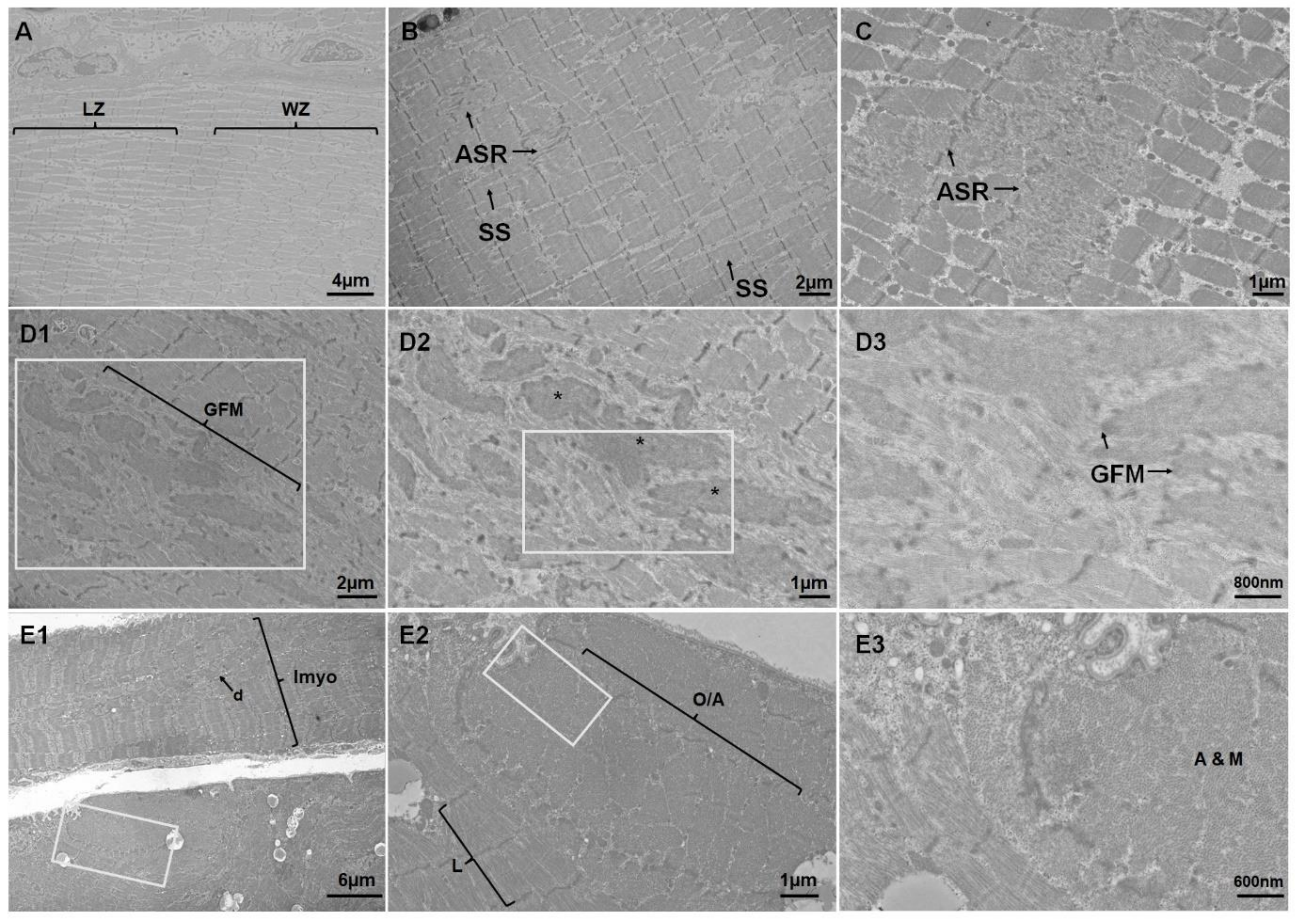
Common abnormal structural features within stage II and stage IV PAD patients. A) Shows compartmentalized regions of linear z-discs (LZ) and wavy Z-discs (WZ) found directly adjacent to each other within a single fiber. B) Shows disorganization and disruption of individual myofibers that appear to have Z-disc smearing and a loss of normal identifiable features (arrows). C) Shows a larger region of sarcomere that appear to have also lost their identifiable features (arrows). D1-D3) Regions that display granular filamentous (GFM) material that is disorganized and electron dense (*) in the intermyofibrillar space. E1-E3) Shows irregular myofibril alignment (Imyo) with myofibrils displaying an oblique/anatomical orientation (O/A) directly adjacent to longitudinally oriented myofibrils. In E3, the lattice structure of the actin and myosin (A&M) filaments is visible next to sarcomeres in longitudinal orientation.

Several regions of both stage II and stage IV patients myofibers displayed large structurally distinct regions of wavy z-discs that contained relatively fewer mitochondria (Figure 4A(Fig. 4)). Additionally, multiple sarcomeres were found to have smeared z-discs or unrecognizable internal sarcomere regions (I-band, A-band, M-line, and H-Zone) (Figure 4 B-C(Fig. 4); ASR). In some stage II and stage IV patients there was a loss of the myofibrillar structure that appeared as granular filamentous material within the IMF space (Figure 4 D1-3(Fig. 4); GFM). These regions contained electron-dense material that was amorphous and lacked distinct structural features (Figure 4 D3(Fig. 4)). No mitochondria were visible within these regions of perturbed myofibril structures. One of the most extreme examples of myofibril disruptions can be viewed in Figure 4 E1-3(Fig. 4). In Figure 4 E1(Fig. 4), the top myofiber displays misaligned Z-discs and intact myofibrils diagonally orientated (Figure 4 E1(Fig. 4); d) that are not uniformly extending the length of the myofiber (Figure 4 E1(Fig. 4); lmyo). Further, in the lower region of Figure 4 E1(Fig. 4), intact myofibrils are present. However, a subregion of the myofiber (magnified in Figure 4 E3(Fig. 4)) shows both sarcomeres in longitudinal orientation as well as the hexagonal lattice structure of the thick and thin filaments typically only visible on oblique or anatomical cross-section. The membrane of the myofiber is unaltered, indicating that the above observation may not be an artifact of tissue processing but a structural deformity that develops with PAD. 

**Figure 5:**
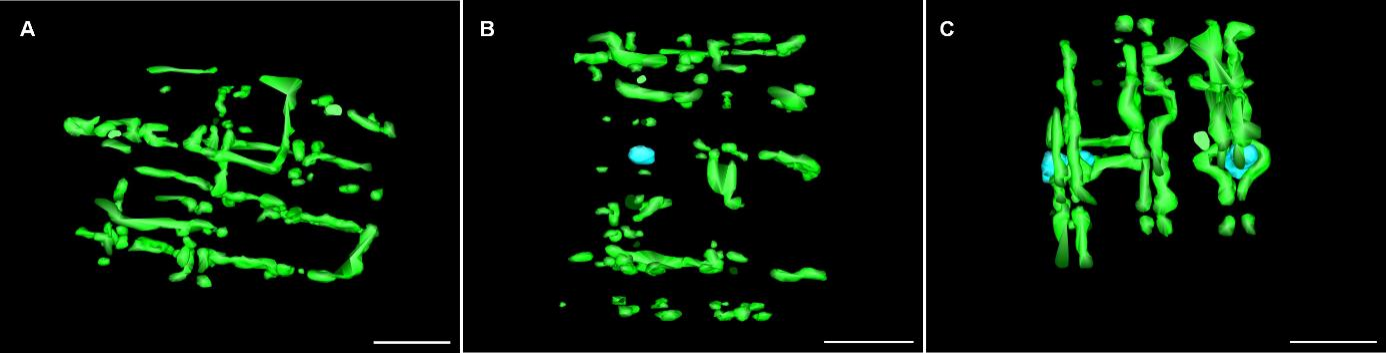
Video still image of the mitochondrial network (green) and IMCL (blue) in control (A), stage II (B), and stage IV (C) PAD patients. The mitochondrial network of Stage II PAD patients seems to be segmented compared to control and stage IV PAD patients.

The mitochondrial three-dimensional models of both the control (Figure 5A(Fig. 5); supplemental Video 1) and the stage IV patient (Figure 5C(Fig. 5), supplemental Video 2) show extensions of the mitochondria transversely across the myofibrils in pairs near the I-band space. These transverse mitochondria columns that appear present in both conditions have occasional branches extending from one column to an adjacent column of mitochondria near a neighboring myofibril. The control condition does appear to have more mitochondria that span the length of individual sarcomeres in the longitudinal direction compared to the stage IV patient. Strikingly, stage II patients appear to have a segmented mitochondrial structure that is not connected (Figure 5B(Fig. 5), supplemental Video 3). The stage IV patient appeared to have IMCL, which had a circular shape and was accompanied by mitochondrial columns that encased the outer border of the IMCLs. The mitochondria that were in direct proximity of these IMCL appeared to have a similar appearance as the outer surface area of the IMCL (like a shell), possibly due to increased IMCL-mitochondria surface area. 

See also the supplementary data.

## Discussion

In this study, we used electron microscopy to quantify cellular alterations in skeletal muscle ultrastructure and to explore their association with the deterioration of muscle metabolism and function in PAD patients. Our analysis identified several ultrastructural modifications in the PAD gastrocnemius, including reduced sarcomere dimensions, alterations in mitochondria number and localization in the z-disc, accumulations of granular filamentous material, myofibrillar disorientation, changes in the quantity of lipid droplets, variations in lipid droplet cross-sectional area, and modifications in mitochondria-lipid droplet contact area. These ultrastructural changes correlated with impaired mitochondrial respiration and increased mitochondrial ROS production. Furthermore, we found that increased content of mitochondria in the muscle tissue (per EM measurements) is associated with improved mitochondrial respiration and reduced oxidative stress. Additionally, we observed a progressive deterioration in our mitochondrial parameters across the stages of PAD. In stage II PAD we observed impaired mitochondrial function and structure while in stage IV PAD we observed further deterioration of mitochondrial function, more pronounced alterations in mitochondria structure and, a unique for stage IV finding of, a decrease in mitochondrial content. Our findings suggest that pathological mitochondria play a key role in the development of the myopathy of PAD and the skeletal muscle dysfunction of PAD patients and represent an important target for therapeutic interventions aimed at improving clinical and functional outcomes in PAD patients. Potential approaches should be instituted early and may include therapies designed to preserve and enhance mitochondrial biogenesis and respiration, improve mitochondrial-lipid droplet interactions, or mitigate oxidative stress.

### Factors beyond blood perfusion contribute to walking limitations in patients with stage II PAD

Walking performance during the 6-minute walking test indicated that stage II patients covered significantly shorter distances than control participants within the same time frame. This impairment was further corroborated by the significantly lower walking distance observed in stage II PAD patients during the graded treadmill test. Similarly, the average 4 m walking speed for the stage II patients was about two-thirds of the control participants. The walking impairments found in stage II PAD, along with the ultrastructural and metabolic alterations in skeletal muscle we demonstrate in this study, indicate that factors beyond just blood perfusion may contribute to the diminished walking ability. This is in line with other studies demonstrating a correlation between parameters of the PAD myopathy like abnormalities of the cytoskeleton, altered myofiber morphology and density, mitochondrial dysfunction and impaired walking ability of patients with PAD (Koutakis et al., 2015[23][24]). Furthermore, several studies in gait biomechanics have demonstrated significant alterations in walking patterns that could explain the walking impairment in PAD (Bapat et al., 2023[4]; Koutakis et al., 2010[22][25], Leutzinger et al., 2022[28]; Rahman et al., 2022[38]; Wurdeman et al., 2012[49]). Stage II patients have been previously shown to have fibrosis and disorganized cytoskeletal structures, which could decrease the efficiency of muscle force transfer during walking (Koutakis et al., 2015[23][24]). The decreased 4 m walking velocity in these patients may be largely due to the damaged intracellular and extracellular structures. In the current study, the average sarcomere M-line lengths for PAD patients were shorter than control participants. The limiting factors present during short walking distances may be caused by intra- and extracellular pathology as they are reflected in PAD myopathy, while longer distances are limited by perfusion. Previous studies, using Focused Ion Beam Scanning Electron Microscopy, have shown that mouse myofibrils exist within a myofibrillar matrix within a muscle fiber (Willingham et al., 2020[48]). Assuming enough sarcomeres were measured in the correct orientation, our finding of a reduction of the average individual sarcomeres size across PAD progression may represent a decrease in the size (or volume) of the myofibrillar matrix and myofiber CSA. However, the direct ultrastructural changes that facilitate changes in myofibrillar hypertrophy and atrophy are still poorly understood and represent a large area of ongoing research (Jorgenson et al., 2020[18]; Roberts et al., 2023[39]; Schwartz, 2019[41]). Decreased sarcomere size would directly contribute to reduced walking ability and decreased isometric force through fewer possible actin and myosin cross bridges. Future studies are warranted to further assess sarcomere morphology to identify whether the myofibril CSA in anatomical cross-section, or sarcomere volumes are reduced with increased disease severity. 

### Ultrastructural aberrations in PAD myopathy: decreased sarcomere size, Z-disc irregularities, filamentous accumulations, and myofibrillar disorientation

In addition to the quantified changes in sarcomere morphology, there were specific regions within the muscle fibers of both stage II and stage IV patients that had irregular Z-disc morphologies such as Z-disc smearing, Z-disc misalignment, and wavy Z-discs. Some participants had noticeable small regions of granular filamentous material present within the IMF space, while others had larger regions of granular filamentous material that occupied the entire field of view. Both qualitative changes in Z-discs and granular filamentous material in the IMF space have been documented previously (Farinon et al., 1984[11]; Hedberg et al., 1988[15]) and confirm the accumulation of such material, likely because of increased damage coupled with failed autophagy, as a significant mechanism in the generation of PAD myopathy. A unique finding in the current study showed myofibers of PAD patients that contained abnormal localization of myofibrils in both anatomical and longitudinal orientations within the same field of view. Such a severe loss of the myofibril orientation within this population would contribute to inadequate force production and may be related to, or caused by, major disruptions of the cytoskeleton or protein degradation systems within the compromised fiber (Aweida et al., 2018[1]; Cohen et al., 2012[8]; Koutakis et al., 2015[23]; Volodin et al., 2017[43]). 

### Progressive deterioration in mitochondrial structure, function, and content in PAD 

The current study observed reduced mitochondrial respiration and increased ROS production across all mitochondrial ETC complexes when comparing control subjects to patients with PAD at different stages. Notably, in the stage II patients, mitochondrial complexes CI+II, III, and IV exhibited decreased respiratory capacity and increased ROS production without concomitant reductions in mitochondrial content. In contrast, Stage IV PAD patients demonstrated reduced respiratory activities across all complexes of the mitochondrial ETC, increased mitochondrial ROS production and significant reductions in the mitochondrial content. These findings suggest that in early PAD, mitochondrial function may be compromised without immediate impact on mitochondrial content while as the disease progresses both function and content of the organelles are compromised. Recent studies employing electron microscopy techniques have shown that skeletal muscle mitochondria in non-pathological conditions form an interconnected grid-like reticular structure (Bakeeva et al., 1978[3]; Glancy et al., 2015[14], 2017[13]; Ogata and Yamasaki, 1985[34]). More recent studies have shown that uncoupling a region of this reticular structure leads to rapid segmentation of the uncoupled region and the interconnectedness of the mitochondrial network may be contingent upon maintenance of mitochondrial membrane potential (Glancy et al., 2015[14], 2017[13]). Qualitative assessments of three-dimensional models of mitochondria in our study revealed fewer instances of mitochondria extending the length of the sarcomere, potentially indicating a more segmented mitochondrial network in PAD-affected muscles. This segmentation could have significant implications for cellular respiration, as it may further limit the rate of ATP production by impeding the diffusion of gases through the myofiber required for mitochondrial respiration. However, studies with larger sample sizes utilizing three-dimensional imaging techniques are necessary to determine quantitative differences and changes in structural patterns of functional and non-functional mitochondria within PAD patients. Such comprehensive analyses would provide a more robust understanding of the relationship between mitochondrial structure, function, and disease progression in PAD.

The analysis of associations between mitochondrial morphological variables and their physiological functions demonstrated several noteworthy relationships. Generally, an increase in the number of mitochondria per EM measurements was associated with enhanced respiration and decreased ROS production across nearly all ETC complexes. This finding suggests a positive correlation between mitochondrial abundance and overall healthy mitochondrial function, with PAD damage on mitochondria reflected in both a decrease in mitochondrial abundance and a decline in mitochondrial function (specifically including ATP production and ROS regulation). Significant associations were also identified between mitochondrial function and both average individual mitochondria CSA and relative mitochondrial area. Specifically, an increase in individual mitochondrial CSA correlated with decreased CII respiration and increased H_2_O_2 _production in CI.2, CI.3, and CIV indicating that the presence of enlarged individual mitochondria in PAD muscles is indicative of physiological dysfunction. Furthermore, an increase in relative mitochondrial area correlated with enhanced respiration for CI+II, CII, CIII, and CIV and decreased ROS production from complexes CI+II, CIII, and CIV. These findings suggest that a greater proportion of mitochondrial area within the muscle tissue is associated with improved mitochondrial respiration and reduced oxidative stress. Taken together, these results demonstrate that mitochondrial structure and function correlate and that when attempting to utilize two dimensional variables associated with changes in mitochondrial structure to infer alterations in mitochondria function, mitochondrial content may be the morphological parameter most reflective of mitochondrial physiological function within skeletal muscle tissue. 

### Intramyocellular lipid dynamics in PAD

IMCL serve as ectopic storage sites of triacylglycerol that interact with mitochondria and regulate lipid metabolism. There are generally two distinct pools of IMCL within skeletal muscle fibers: the subsarcolemmal and IMF IMCLs. Sedentary lifestyles and overconsumption of fats lead to an increase in IMCL within the subsarcolemmal region and are associated with type II diabetes (Bergman et al., 2012[5]; Crane et al., 2010[9]; Nielsen et al., 2010[32]). In our study, the CSA of IMF IMCL was significantly increased within the stage IV patients. This increase could be attributed to a reduction in lipolysis, physical inactivity, an increase in dietary fat intake, or a combination of these factors. Prior research has shown decreases in total IMCL content and IMF IMCL cell fraction following metabolic stress from exhaustive exercise (Badin et al., 2013[2]; Koh et al., 2017[20]). Paradoxically, exercise typically elicits an anaerobic response in PAD patients due to ischemia within the leg muscles, potentially dampening the overall lipolysis occurring within this tissue and contributing to IMCL growth. Interestingly, exercise has also been shown to increase the length of contact area between mitochondria and lipid droplets within normal, obese/non-diabetic, and obese/diabetic individuals (de Almeida et al., 2023[10]). Additionally, the magnitude of mitochondria-IMCL interface length before exercise was also correlated with fat oxidation rate during exercise regardless of obesity or diabetes status (de Almeida et al., 2023[10]). The authors suggest that this increase in contact between the IMCL and mitochondria may occur to facilitate a faster rate of lipolysis for ATP production under exercise-induced metabolic stress (de Almeida et al., 2023[10]). Our study showed an increase in IMCL-mitochondria contact length in the stage IV PAD patients compared to controls in the rested state, suggesting that these patients experience intramuscular metabolic stress due to ischemia. This enlargement of IMCL may indicate either a malfunction of the metabolic process of fatty acid oxidation or that skeletal muscle fiber fatty acid uptake overtakes fatty acid oxidation (Morales et al., 2017[30]). 

### Intracellular swelling in PAD skeletal muscle

Within the PAD condition, irrespective of disease stage, we observed increases in sarcoplasmic area indicative of intracellular swelling. Typically, intracellular swelling is accompanied by an impaired ability to regulate ion transport across cellular membranes. Theoretically, inhibition of Na^+^-K^+^ ATPase pumps (NKA) could facilitate myofibril swelling due to dysregulated Na^+^ flux. PAD patients experience ischemia, which can limit ATP production and increase H^+^ concentrations within the muscle fiber. Under these conditions, Na^+^-H^+^ exchangers may become activated in response to decreased pH, thereby facilitating the influx of Na^+ ^(Juel, 1998[19]). The reduction in ATP would also lead to an inhibition of NKA activity and could lead to an influx of Na^+^ until reperfusion occurs. Foundational work on isolated skeletal muscle from rats showed that inhibition of NKA with ouabain, severely decreased the excitability of the skeletal muscle, resulting in rapid reductions in force production, significant increases in intracellular Na^+, ^and a more positive resting membrane potential (Murphy and Clausen, 2007[31]). Taken together, NKA inhibition could explain the observed changes in the sarcoplasmic areas and contribute to the decreased contractile function found in PAD patients. Interestingly, to the authors knowledge, there has yet to be a study assessing differences in resting membrane potentials, resting Na^+ ^concentration, or alterations in Na^+ ^transport within the skeletal muscle of PAD patients before or after a cycle of ischemia-reperfusion and this represents a significant gap in our understanding of the pathophysiological mechanisms underlying muscle dysfunction in PAD.

This study is not without its limitations. All the images analyzed within this study were collected when the participants were at rest and may not reflect certain changes that occur after different modalities, such as exercise or revascularization. Further, two-dimensional micrograph analysis using TEM provides insight into the cellular structure of a specific location of the myofiber and may miss certain structural alterations that occur within other regions of this tissue. Future studies would benefit from sampling alternative regions of the same muscle and incorporating samples from other muscle groups to see if these changes are constant across muscle groups in each disease stage. 

In conclusion, this study provides a comprehensive analysis of the ultrastructural basis of PAD-associated myopathy, emphasizing the critical role of mitochondrial abnormalities. Our data demonstrate a progressive deterioration of both muscle function and structure in patients with PAD, that is evident in stage II and becomes worse in stage IV of the disease. These findings suggest that stage II PAD represents a critical phase for implementing therapeutic interventions aimed at improving mitochondrial function and structure, before extensive mitochondrial damage occurs, and underscore the importance of early detection and intervention in PAD. Potential treatment approaches may include therapies designed to: enhance mitochondrial biogenesis and respiratory capacity, improve mitochondrial-lipid droplet interactions, or mitigate oxidative damage. It is possible that such interventions, when applied early in the course of the disease, could ameliorate the myopathy of PAD and this in turn has the potential to improve clinical and functional outcomes in PAD patients. 

## Declaration

### Acknowledgments

We thank all study participants for their invaluable contribution to this research study.

### Funding

This work was supported by the National Institute on Aging of the National Institutes of Health under Award Numbers R01AG064420 (P.K.) and R01AG077803 (I.I.P). The content is solely the responsibility of the authors and does not necessarily represent the official views of the National Institutes of Health. Additionally, the study was supported by the American Heart Association grant #17SDG33630088 (P.K.) and the San Antonio Medical Foundation (P.K., D.M.).

### Conflict of interest

The authors declare no conflict of interest. The funding agencies had no role in the design of the study; in the collection, analyses, or interpretation of data; in the writing of the manuscript, or in the decision to publish the results.

### Data availability 

The original contributions presented in the study are included in the article; further inquiries can be directed to the corresponding author.

## Supplementary Material

Video 1 Control

Video 2 Stage II

Video 3 Stage IV

Supplementary data

## Figures and Tables

**Table 1:**
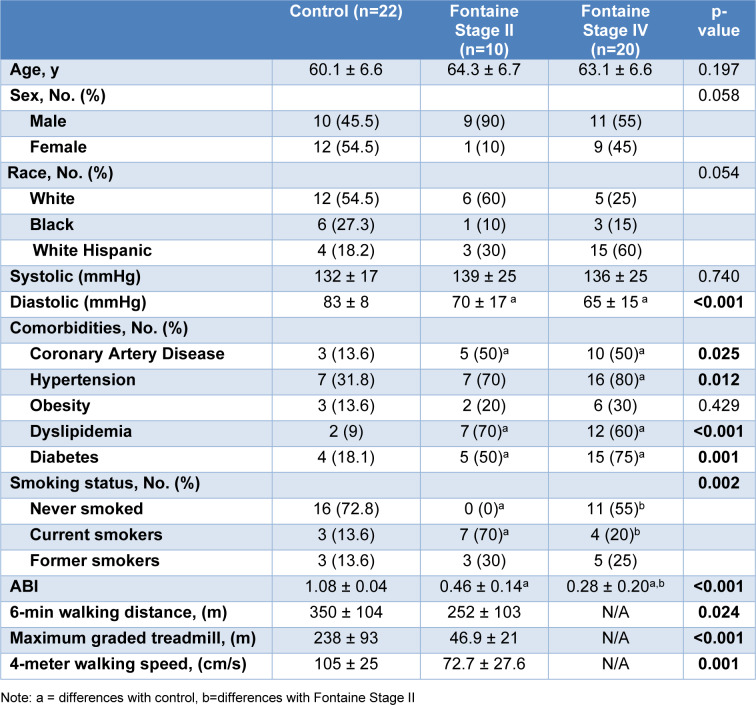
Patient demographics

**Table 2:**
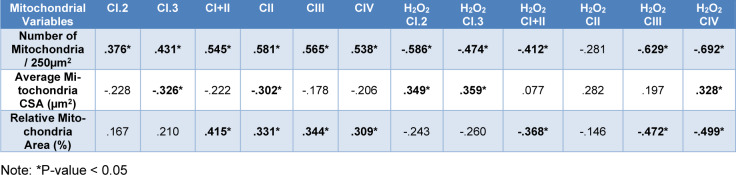
Correlations of mitochondrial morphology with mitochondrial function
