# Supplementary material for: Ultrastructural alterations and mitochondrial dysfunction in skeletal muscle of peripheral artery disease patients: implications for early therapeutic interventions
Source: EXCLI J. 2024 Oct 7;23:1208–25. doi: 10.17179/excli2024-7592 (PMC11579521; doi:10.17179/excli2024-7592)
Supplement: Supplementary data [file EXCLI-23-1208-s-004.pdf]

EXCLI Journal 2024;23:1208-1225 – ISSN 1611-2156
Received: July 03, 2024, accepted: September 06, 2024, published: October 07, 2024

Original article:

ULTRASTRUCTURAL ALTERATIONS AND MITOCHONDRIAL DYSFUNCTION IN SKELETAL MUSCLE OF PERIPHERAL ARTERY DISEASE PATIENTS: IMPLICATIONS FOR EARLY THERAPEUTIC INTERVENTIONS

Dylan Wilburn[a], Emma Fletcher[b], Evlampia Papoutsi[b], William T. Bohannon[c], Gleb Haynatzki[d], Bernd Zechmann[e], Yuqian Tian[f], Iraklis I. Pipinos[f], Dimitrios Miserlis[g], Panagiotis Koutakis[b,h,*]

[a] Department of Cell Biology, University of Texas Southwestern Medical Center, Dallas, TX, USA
[b] Department of Public Health, University of West Florida, Pensacola, FL, USA
[c] Department of Surgery, Baylor Scott & White Medical Center, Temple, TX, USA
[d] Department of Biostatistics, University of Nebraska Medical Center, Omaha, NE, USA
[e] Center for Microscopy and Imaging, Baylor University, Waco, TX, USA
[f] Department of Surgery, University of Nebraska Medical Center, Omaha, NE, USA
[g] Department of Surgery, University of Texas Dell Medical School, Austin, TX, USA
[h] Department of Biology, Hal Marcus College of Science and Engineering, Pensacola, FL, USA

* **Corresponding author:** Panagiotis Koutakis, Department of Public Health, University of West Florida, Pensacola, FL, USA. Tel.: +1 850-474-3191, 11000 University Parkway, Building 38, Room 106A, Pensacola, FL 32514. E-mail: pkoutakis@uwf.edu

https://dx.doi.org/10.17179/excli2024-7592

This is an Open Access article distributed under the terms of the Creative Commons Attribution License (http://creativecommons.org/licenses/by/4.0/).

EXCLI Journal 2024;23:1208-1225 – ISSN 1611-2156
Received: July 03, 2024, accepted: September 06, 2024, published: October 07, 2024

Raw data concerning **Table 1:** Patient demographics

ID	AGE	Sex, M=male, F=female	RACE, W=white, B=black, WH=white hispanic	Diastolic (mmHg)	Systolic (mmHg)	Coronary artery disease, 0=no, 1=yes	Hypertension, 0=no, 1=yes	Obesity, 0=no, 1=yes	Dyslipidemia, 0=no, 1=yes	Smoking status, 0=never smoked, 1=current smoker, 2=former smoker	Diabetes, 0=no, 1=yes
**1001**	52.00	F	WH	141	60.00	0.00	1.00	0.00	1.00	1.00	1.00
**1002**	60.00	F	W	129	80.00	0.00	1.00	1.00	0.00	1.00	0.00
**1003**	52.00	F	W	123	85.00	0.00	0.00	0.00	0.00	0.00	0.00
**1004**	68.00	M	WH	149	66.00	1.00	1.00	0.00	0.00	1.00	1.00
**1005**	66.00	M	WH	169	84.00	0.00	1.00	0.00	0.00	1.00	1.00
**1006**	60.00	M	WH	126	86.00	1.00	0.00	0.00	1.00	0.00	0.00
**1007**	60.00	M	W	106	67.00	1.00	1.00	0.00	1.00	1.00	0.00
**1008**	64.00	F	WH	143	54.00	0.00	0.00	0.00	0.00	0.00	1.00
**1009**	58.00	F	WH	115	76.00	0.00	0.00	0.00	1.00	0.00	1.00
**1010**	71.00	F	B	123	75.00	0.00	0.00	0.00	0.00	0.00	0.00
**1011**	74.00	M	W	149	53.00	1.00	1.00	0.00	1.00	2.00	1.00
**1012**	68.00	M	WH	95	50.00	1.00	1.00	0.00	1.00	0.00	1.00
**1013**	71.00	F	B	103	59.00	0.00	0.00	0.00	0.00	0.00	0.00
**1014**	65.00	F	B	140	42.00	0.00	1.00	1.00	1.00	0.00	1.00
**1015**	66.00	M	W	142	80.00	1.00	1.00	0.00	0.00	1.00	1.00
**1016**	58.00	F	W	126	89.00	0.00	0.00	0.00	0.00	0.00	0.00
**1017**	60.00	F	WH	119	54.00	0.00	1.00	0.00	1.00	2.00	1.00
**1018**	59.00	F	W	146	83.00	0.00	0.00	0.00	0.00	0.00	0.00
**1019**	67.00	F	W	131	41.00	1.00	1.00	1.00	0.00	0.00	1.00
**1020**	66.00	F	W	121	79.00	0.00	1.00	0.00	0.00	2.00	0.00

**1021**	54.00	M	W	139	89.00	0.00	0.00	0.00	0.00	0.00	0.00
**1022**	63.00	M	W	120	71.00	0.00	0.00	0.00	1.00	1.00	1.00
**1023**	65.00	M	W	129	92.00	0.00	1.00	0.00	0.00	2.00	1.00
**1024**	70.00	F	WH	176	75.00	1.00	1.00	0.00	1.00	0.00	1.00
**1025**	71.00	M	WH	144	56.00	1.00	0.00	1.00	0.00	2.00	0.00
**1026**	67.00	F	B	139	55.00	0.00	1.00	1.00	0.00	1.00	0.00
**1027**	63.00	F	WH	116	80.00	1.00	1.00	0.00	1.00	2.00	0.00
**1028**	47.00	M	WH	115	68.00	1.00	1.00	1.00	1.00	0.00	1.00
**1029**	51.00	M	W	124	88.00	1.00	1.00	0.00	1.00	1.00	0.00
**1030**	59.00	M	WH	148	68.00	0.00	0.00	1.00	0.00	0.00	1.00
**1031**	67.00	F	WH	154	54.00	0.00	1.00	1.00	1.00	0.00	1.00
**1032**	53.00	M	B	128	86.00	0.00	0.00	1.00	0.00	0.00	0.00
**1033**	72.00	M	W	119	62.00	1.00	1.00	0.00	1.00	2.00	0.00
**1034**	56.00	F	W	169	98.00	0.00	1.00	0.00	0.00	2.00	0.00
**1035**	53.00	M	W	119	88.00	0.00	0.00	0.00	0.00	1.00	0.00
**1036**	60.00	M	WH	164	48.00	1.00	1.00	0.00	1.00	2.00	1.00
**1037**	60.00	M	WH	161	70.00	0.00	1.00	0.00	1.00	0.00	1.00
**1038**	58.00	M	B	140	82.00	1.00	1.00	0.00	0.00	0.00	1.00
**1039**	54.00	M	W	142	83.00	0.00	1.00	1.00	0.00	0.00	0.00
**1040**	71.00	M	B	149	75.00	0.00	1.00	0.00	1.00	1.00	0.00
**1041**	72.00	F	B	144	99.00	0.00	0.00	0.00	0.00	0.00	0.00
**1042**	65.00	M	W	144	44.00	1.00	1.00	1.00	1.00	1.00	1.00
**1043**	57.00	F	W	116	79.00	0.00	0.00	0.00	0.00	0.00	0.00
**1044**	65.00	M	W	144	90.00	0.00	1.00	0.00	0.00	0.00	1.00
**1045**	67.00	M	B	96	67.00	1.00	0.00	0.00	0.00	0.00	0.00
**1046**	64.00	M	WH	109	97.00	1.00	1.00	0.00	1.00	0.00	1.00
**1047**	61.00	F	W	124	73.00	0.00	1.00	0.00	0.00	2.00	1.00
**1048**	62.00	M	W	161	77.00	0.00	0.00	0.00	0.00	1.00	0.00
**1049**	53.00	M	WH	124	83.00	0.00	0.00	0.00	0.00	0.00	0.00
**1050**	71.00	F	WH	111	74.00	0.00	1.00	0.00	0.00	0.00	0.00

EXCLI Journal 2024;23:1208-1225 – ISSN 1611-2156
Received: July 03, 2024, accepted: September 06, 2024, published: October 07, 2024

**1051**	60.00	M	B	168	90.00	0.00	0.00	0.00	0.00	2.00	0.00
**1052**	56.00	M	W	194	101.00	0.00	0.00	0.00	1.00	1.00	0.00

EXCLI Journal 2024;23:1208-1225 – ISSN 1611-2156
Received: July 03, 2024, accepted: September 06, 2024, published: October 07, 2024

Figure 1 associated data. Raw data for relative mitochondrial area (%), number of mitochondria per 250 μm^2, mitochondria cross sectional area (CSA), and mitochondria per Z disc.

ID	Relative Mitochondria Area (%)	Average Mitochondria CSA	Number of Mitochondria / 250 μm^2	Mitochondria / Zdisc
**1001**	7.327	0.285	64.220	1.088
**1002**	1.283	0.066	48.360	0.332
**1003**	2.783	0.115	60.400	0.654
**1004**	2.954	0.046	161.550	1.563
**1005**	4.995	0.081	153.555	1.230
**1006**	4.411	0.207	53.266	0.447
**1007**	3.725	0.194	47.895	0.482
**1008**	4.328	0.093	116.757	1.067
**1009**	5.141	0.171	75.112	0.597
**1010**	5.189	0.094	137.644	1.256
**1011**	3.846	0.188	51.137	0.404
**1012**	2.099	0.041	128.609	0.961
**1013**	5.572	0.113	123.353	1.139
**1014**	3.680	0.101	90.647	0.838
**1015**	4.862	0.099	123.003	1.696
**1016**	1.523	0.196	19.401	0.137
**1017**	6.529	0.101	162.399	1.259
**1018**	1.927	0.065	74.292	0.815
**1019**	3.848	0.089	107.945	0.938
**1020**	5.293	0.113	117.396	1.345
**1021**	2.480	0.103	60.207	0.500
**1022**	3.736	0.053	176.969	1.340
**1023**	1.332	0.106	31.539	0.290
**1024**	3.866	0.144	66.933	0.389
**1025**	1.178	0.088	33.291	0.217
**1026**	4.346	0.070	155.943	1.353
**1027**	2.017	0.136	37.146	0.279
**1028**	2.870	0.053	136.669	0.929
**1029**	2.605	0.074	87.434	0.734
**1030**	2.026	0.186	27.269	0.277
**1031**	2.356	0.204	28.847	0.248
**1032**	7.274	0.102	178.571	1.422
**1033**	3.714	0.078	119.683	0.947
**1034**	0.052	0.005	25.582	0.215
**1035**	1.412	0.174	20.300	0.163
**1036**	3.993	0.151	66.232	0.566
**1037**	3.926	0.067	146.131	1.279

EXCLI Journal 2024;23:1208-1225 – ISSN 1611-2156
Received: July 03, 2024, accepted: September 06, 2024, published: October 07, 2024

**1038**	3.691	0.108	85.467	0.658
**1039**	0.940	0.042	56.144	0.404
**1040**	0.665	0.148	11.214	0.080
**1041**	0.007	0.000	97.421	0.820
**1042**	0.018	0.001	35.043	0.337
**1043**	5.669	0.119	119.148	0.960
**1044**	2.538	0.057	110.693	1.432
**1045**	4.291	0.080	134.585	1.005
**1046**	1.825	0.056	81.904	1.137
**1047**	2.983	0.058	127.909	0.966
**1048**	5.203	0.066	198.346	1.497
**1049**	3.948	0.077	128.259	0.961
**1050**	4.241	0.061	174.166	1.702
**1051**	2.762	0.104	66.232	0.507
**1052**	3.458	0.078	110.387	0.799

Figure 2 associated data. Individual data for mitochondrial respiration (JO_2) and hydrogen peroxide production (H_2O_2).

Data of JO_2 for all the patients

ID	Complex CI.2	Complex I.3	Complex I+II	Complex II	Complex III	Complex IV
**1001**	4.73	23.59	22.91	7.26	73.87	84.10
**1002**	0.92	4.13	5.08	2.56	32.55	20.56
**1003**	4.98	8.40	4.46	4.54	65.98	25.73
**1004**	5.74	25.54	12.66	6.91	120.14	113.97
**1005**	3.69	11.20	8.45	6.85	15.91	108.81
**1006**	1.01	1.99	6.36	2.11	17.77	77.88
**1007**	2.66	4.88	1.88	3.55	41.05	41.29
**1008**	2.96	11.55	5.38	8.21	15.53	14.04
**1009**	2.85	6.70	6.34	4.28	14.93	51.56
**1010**	0.85	7.69	9.57	8.18	119.37	68.47
**1011**	1.97	5.14	0.82	3.26	20.30	74.26
**1012**	4.16	10.07	14.56	10.52	91.04	163.40
**1013**	4.98	20.47	21.56	4.55	60.55	85.56
**1014**	6.31	15.11	15.52	5.82	186.06	54.78
**1015**	6.12	10.74	10.91	5.12	152.63	141.29
**1016**	1.11	3.10	6.35	3.25	9.72	28.46
**1017**	2.12	6.00	7.56	8.47	70.16	56.85
**1018**	2.66	4.85	10.55	3.13	55.55	48.55
**1019**	2.96	11.55	8.57	8.21	25.46	14.04
**1020**	3.55	16.55	18.56	10.56	90.56	80.56
**1021**	2.24	6.17	12.96	5.57	8.89	85.57
**1022**	4.14	12.68	14.43	10.55	100.47	125.00
**1023**	1.53	2.57	4.57	1.81	12.80	73.99
**1024**	5.75	1.17	2.69	4.74	38.11	24.51
**1025**	1.45	1.87	1.99	1.83	5.86	43.53
**1026**	4.85	6.61	6.56	5.07	64.09	92.96
**1027**	1.70	6.20	4.78	3.58	20.32	41.06
**1028**	3.46	10.95	1.68	5.91	58.00	220.00
**1029**	0.88	0.95	6.45	1.60	18.46	74.75
**1030**	2.84	5.09	4.78	2.90	9.66	13.87
**1031**	3.39	6.33	3.73	4.26	42.05	76.43
**1032**	2.55	9.54	12.55	8.55	125.55	135.55
**1033**	3.14	10.17	14.60	11.55	45.56	210.00
**1034**	1.71	7.06	5.77	3.52	46.38	75.79
**1035**	2.74	6.95	5.77	4.56	14.18	33.27
**1036**	5.24	12.28	12.04	4.37	157.29	47.45
**1037**	2.29	8.71	9.97	12.56	78.95	82.45
**1038**	0.79	1.31	12.96	2.26	38.89	124.63

EXCLI Journal 2024;23:1208-1225 – ISSN 1611-2156
Received: July 03, 2024, accepted: September 06, 2024, published: October 07, 2024

**1039**	2.44	5.94	4.77	3.82	22.53	77.30
**1040**	4.05	8.62	7.56	3.59	4.89	33.28
**1041**	1.88	10.89	6.52	4.81	15.85	72.55
**1042**	3.24	7.17	9.82	6.09	21.94	63.45
**1043**	2.93	7.76	9.07	4.85	29.04	173.81
**1044**	4.55	15.14	17.55	8.57	110.55	95.47
**1045**	3.94	13.07	7.91	7.03	14.57	66.60
**1046**	3.21	8.14	15.20	5.44	25.41	70.31
**1047**	3.46	14.99	6.92	3.94	85.55	100.66
**1048**	2.01	5.14	12.05	3.16	67.61	88.99
**1049**	6.55	22.20	22.64	8.34	190.22	139.64
**1050**	5.69	10.16	10.34	9.19	92.58	99.21
**1051**	2.22	6.65	9.14	12.22	31.48	45.65
**1052**	2.26	8.53	23.16	3.62	78.60	107.80

EXCLI Journal 2024;23:1208-1225 – ISSN 1611-2156
Received: July 03, 2024, accepted: September 06, 2024, published: October 07, 2024

Individual data of H_2O_2 for all the patients

ID	Complex CI.2	Complex I.3	Complex I+II	Complex II	Complex III	Complex IV
**1001**	0.067	0.106	0.012	0.301	0.376	0.686
**1002**	0.156	0.165	0.201	0.062	1.061	1.285
**1003**	0.031	0.263	0.157	0.900	0.852	1.400
**1004**	0.049	0.006	0.004	0.060	0.261	0.463
**1005**	0.035	0.156	0.102	0.209	1.012	1.200
**1006**	0.265	0.743	0.030	0.111	0.860	2.800
**1007**	0.125	0.101	0.046	0.133	0.914	1.676
**1008**	0.052	0.014	0.102	0.309	1.138	0.925
**1009**	0.113	0.608	0.049	0.354	1.460	2.465
**1010**	0.055	0.150	0.069	0.425	0.598	0.210
**1011**	0.078	0.084	0.115	1.403	1.788	3.406
**1012**	0.005	0.343	0.048	1.052	0.036	0.491
**1013**	0.047	0.100	0.029	0.641	0.387	0.533
**1014**	0.053	0.173	0.025	0.485	0.472	0.409
**1015**	0.019	0.022	0.013	0.074	0.427	0.707
**1016**	0.151	0.457	0.089	0.431	1.256	4.671
**1017**	0.120	0.137	0.091	0.803	0.360	0.370
**1018**	0.172	0.570	0.215	0.800	1.976	1.856
**1019**	0.217	0.740	0.267	0.650	0.868	1.259
**1020**	0.100	0.155	0.133	0.144	0.100	0.500
**1021**	0.134	1.970	0.285	0.750	0.769	1.766
**1022**	0.012	0.008	0.005	0.075	0.118	0.237
**1023**	0.095	0.112	0.196	0.060	1.256	1.316
**1024**	0.114	0.146	0.106	0.644	2.725	3.667
**1025**	0.162	0.130	0.096	0.274	2.276	1.258
**1026**	0.045	0.023	0.027	0.113	0.279	0.401
**1027**	0.243	0.124	0.106	0.625	6.014	4.845
**1028**	0.051	0.116	0.393	0.501	0.917	0.928
**1029**	0.154	0.836	0.280	1.273	5.094	1.657
**1030**	0.108	0.101	0.078	0.419	1.324	3.532
**1031**	0.206	0.207	0.146	1.008	3.630	5.481
**1032**	0.059	0.511	0.035	0.253	0.512	0.239
**1033**	0.036	0.399	0.055	0.083	0.980	2.183
**1034**	0.357	0.234	0.312	1.133	6.453	6.265
**1035**	0.072	0.108	0.110	0.707	1.638	1.788
**1036**	0.071	0.272	0.041	0.817	0.568	0.368
**1037**	0.014	0.132	0.076	0.154	1.544	1.333
**1038**	0.076	0.104	0.102	0.330	1.777	0.928
**1039**	0.153	0.646	0.208	0.799	3.718	1.583
**1040**	0.159	0.415	0.305	0.550	1.584	2.356

EXCLI Journal 2024;23:1208-1225 – ISSN 1611-2156
Received: July 03, 2024, accepted: September 06, 2024, published: October 07, 2024

**1041**	0.107	0.163	0.103	0.128	1.536	1.035
**1042**	0.085	0.171	0.101	0.308	3.160	1.289
**1043**	0.069	0.062	0.063	0.135	0.108	0.200
**1044**	0.083	0.122	0.027	0.147	0.476	0.267
**1045**	0.018	0.018	0.101	0.053	1.184	0.516
**1046**	0.050	0.250	0.100	0.203	1.900	1.100
**1047**	0.001	0.002	0.003	0.046	0.047	0.181
**1048**	0.052	0.100	0.100	0.304	0.952	0.925
**1049**	0.043	0.059	0.015	0.153	0.399	0.186
**1050**	0.039	0.079	0.037	0.348	0.520	0.397
**1051**	0.186	0.169	0.103	0.483	1.983	1.638
**1052**	0.025	0.094	0.104	0.239	0.390	0.457

EXCLI Journal 2024;23:1208-1225 – ISSN 1611-2156
Received: July 03, 2024, accepted: September 06, 2024, published: October 07, 2024

Figure 3 associated data. Individual data for relative myofibril area (%), relative lipid droplet area (%), average lipid droplet cross sectional area (µm), number of Z Discs/250 µm², number of lipid droplets per 250 µm², relative sarcoplasmic area (%), sarcomere M-line length (µm), IMCL-mitochondria contact length (µm).

ID	Relative Myofibril Area (%)	Relative lipid droplet area (%)	Average lipid droplet cross sectional area (µm)	Number of Z Discs/250µm²	Number of Lipid Droplets/ 250µm²	Relative Sarcoplasmic Area (%)	Sarcomere M-line length (µm)	MCL-mitochondria contact length (µm)
**1001**	67.5	0.606	0.244	59.0	6.21	24.58	1.361	0.705
**1002**	83.0	0.149	0.118	145.8	3.15	15.56	0.821	0.186
**1003**	71.6	1.975	0.381	124.8	12.97	21.40	0.718	0.452
**1004**	81.4	0.332	0.263	103.4	3.15	15.27	0.962	0.275
**1005**	65.2	3.057	0.287	54.6	26.60	22.95	0.882	0.446
**1006**	61.7	1.126	0.237	125.8	11.88	32.03	0.726	0.389
**1007**	58.3	1.395	0.499	99.3	6.99	36.56	0.814	0.335
**1008**	74.7	0.131	0.235	109.4	1.40	20.87	0.774	0.307
**1009**	78.0	0.086	0.617	124.2	0.35	20.51	0.848	0.279
**1010**	69.7	0.045	0.159	109.6	0.70	25.07	0.804	0.156
**1011**	64.7	0.786	0.491	119.1	4.01	31.91	0.397	0.360
**1012**	85.4	0.161	0.144	133.9	2.80	12.31	1.002	0.209
**1013**	68.3	0.274	0.280	108.3	2.45	25.82	0.876	0.225
**1014**	79.0	0.367	0.262	108.2	3.50	16.98	0.969	0.379
**1015**	70.9	3.709	0.456	72.5	20.33	20.55	0.915	0.380
**1016**	73.8	0.867	0.773	139.8	2.80	24.69	0.662	0.542
**1017**	70.1	1.224	0.220	128.9	13.91	22.16	0.851	0.317
**1018**	66.2	1.371	0.232	92.4	14.74	29.64	0.919	0.212
**1019**	75.2	0.146	0.131	120.4	2.80	22.17	0.906	0.195
**1020**	73.3	0.195	0.198	87.3	2.45	21.17	1.172	0.280
**1021**	71.2	0.292	0.298	91.1	2.45	26.57	0.989	0.226
**1022**	73.8	0.088	0.158	132.1	1.40	22.39	0.733	0.090
**1023**	71.3	0.515	0.459	108.6	2.80	26.86	0.732	0.405
**1024**	71.6	2.169	0.737	172.1	7.36	22.39	0.598	0.377
**1025**	51.1	2.414	0.718	119.1	8.41	42.10	0.647	0.414

**1026**	76.0	0.023	0.017	115.3	0.26	19.67	0.934	0.148
**1027**	74.3	1.730	1.234	133.2	3.50	21.97	0.599	0.236
**1028**	73.9	0.548	0.261	147.2	5.26	22.65	0.847	0.226
**1029**	78.1	0.094	0.167	116.1	1.41	19.49	1.205	0.382
**1030**	77.3	1.294	1.028	98.6	3.15	19.33	0.813	0.354
**1031**	76.3	0.445	1.071	141.7	1.04	21.76	0.703	0.040
**1032**	73.9	0.116	0.050	125.5	5.80	18.72	0.845	0.171
**1033**	76.9	0.594	0.157	126.4	9.48	18.75	1.175	0.353
**1034**	65.3	0.133	0.068	118.8	4.91	34.55	0.661	0.572
**1035**	75.0	0.121	0.286	139.1	1.06	23.93	0.696	0.479
**1036**	72.6	0.168	0.300	117.0	1.40	23.24	1.051	0.681
**1037**	75.0	0.519	0.154	114.2	8.41	20.52	1.130	0.297
**1038**	66.7	0.196	0.228	129.8	2.15	29.45	0.734	0.247
**1039**	64.5	0.067	0.155	126.6	1.07	31.55	0.764	0.456
**1040**	66.1	0.041	0.030	115.1	3.38	29.97	0.906	0.273
**1041**	78.3	0.033	0.059	118.8	1.40	21.63	0.905	0.123
**1042**	73.4	0.041	0.015	104.1	7.01	26.52	0.887	0.873
**1043**	65.8	0.157	0.187	124.1	2.10	28.32	0.772	0.228
**1044**	75.4	0.107	0.096	77.3	2.78	21.91	0.987	0.023
**1045**	73.1	0.573	0.158	133.9	9.09	22.03	0.777	0.141
**1046**	69.3	0.219	0.312	72.1	1.76	28.64	0.705	0.495
**1047**	80.2	0.454	0.202	132.5	5.61	16.37	0.941	0.299
**1048**	69.2	1.601	0.190	132.5	21.03	24.00	0.602	0.259
**1049**	69.3	0.246	0.195	133.5	3.15	26.53	0.820	0.216
**1050**	79.0	0.216	0.128	102.3	4.21	16.54	1.025	0.252
**1051**	68.8	0.362	0.645	130.7	1.40	28.03	0.667	0.152
**1052**	75.5	0.409	0.224	138.1	4.56	20.64	0.705	0.353
